# Interprofessional education day 2019 – a qualitative participant evaluation 

**DOI:** 10.3205/zma001573

**Published:** 2022-11-15

**Authors:** Stefan Gysin, Marion Huber, Emanuel Feusi, Andreas Gerber-Grote, Claudia M. Witt

**Affiliations:** 1 University of Lucerne, Department of Health Sciences and Medicine, Lucerne, Switzerland; 2 ZHAW Zurich University of Applied Sciences (ZHAW), Department of Health, Zurich, Switzerland; 3 University Hospital Zurich and University of Zurich, Institute for Complementary and Integrative Medicine, Zurich, Switzerland

**Keywords:** interprofessionality, interprofessional education, medicine, health professions, interprofessional collaboration, interprofessional competencies

## Abstract

**Objective:**

Interprofessional education (IPE) is when two or more students from different professions learn with, from, and about each other to improve collaboration and quality of healthcare. In October 2019, a first interprofessional education (IPE) day was held in the canton of Zurich with the aim of teaching interprofessional skills to participating students.

**Methodology:**

The IPE day was developed by an interprofessional team of students. After a short introduction, the roles and tasks of the professional groups involved were discussed. This was followed by two case studies with simulation persons and reflection rounds. For the evaluation of the day, 15 semi-structured interviews with students and lecturers were conducted and qualitatively evaluated by means of thematic analysis.

**Results:**

The students and lecturers had a very positive experience of the IPE day. Especially the participation of medical and pharmacy students, the practical case studies with simulation persons and the informal exchange during the breaks were appreciated. There was room for improvement in the development of role models. Through an open attitude and good communication, the students learned to know and appreciate the competencies of the other professional groups. All those interviewed wished for more interprofessional teaching opportunities and the students felt encouraged to apply what they had learned in their later professional practice.

**Conclusion:**

The IPE day could be carried out successfully and the didactic concept worked largely well. The evaluation provided subjective evidence that the students were able to improve the interprofessional competencies of teamwork, communication, openness, appreciation and reflectiveness. In the future, the IPE day should be anchored in the curricula.

## Zusammenfassung

**Zielsetzung:**

Von interprofessioneller Ausbildung (engl. Interprofessional Education, IPE) wird gesprochen, wenn zwei oder mehr Studierende verschiedener Berufsgruppen mit-, von- und übereinander lernen, um die Zusammenarbeit und die Qualität der Gesundheitsversorgung zu verbessern. Im Oktober 2019 fand ein erster interprofessioneller Ausbildungstag (IPE Tag) im Kanton Zürich statt mit dem Ziel, den teilnehmenden Studierenden interprofessionelle Kompetenzen zu vermitteln.

**Methodik:**

Der IPE Tag wurde von einem interprofessionellen Studierendenteam entwickelt. Nach einer kurzen Einführung wurden die Rollen und Aufgaben der beteiligten Berufsgruppen diskutiert. Anschließend gab es zwei Fallbeispiele mit Simulationspersonen und Reflexionsrunden. Zur Evaluation des Tages wurden 15 semi-strukturierte Interviews mit Studierenden und Dozierenden geführt und mittels Thematischer Analyse qualitativ ausgewertet.

**Ergebnisse:**

Die Studierenden und Dozierenden haben den IPE Tag sehr positiv erlebt. Vor allem die Teilnahme der Medizin- und Pharmaziestudierenden, die praktischen Fallbeispiele mit Simulationspersonen und der informelle Austausch in den Pausen wurden geschätzt. Verbesserungspotenzial gab es bei der Erarbeitung der Rollenbilder. Durch eine offene Haltung und gute Kommunikation lernten die Studierenden die Kompetenzen der anderen Berufsgruppen kennen und schätzen. Alle interviewten Personen wünschten sich mehr interprofessionelle Lehrangebote und die Studierenden fühlten sich bestärkt, das Gelernte in der späteren Berufspraxis umzusetzen.

**Schlussfolgerung:**

Der IPE Tag konnte erfolgreich durchgeführt werden und das didaktische Konzept hat weitgehend gut funktioniert. Die Evaluation lieferte subjektive Hinweise, dass die Studierenden die interprofessionellen Kompetenzen Teamfähigkeit, Kommunikation, Offenheit, Wertschätzung und Reflexionsfähigkeit verbessern konnten. In Zukunft soll der IPE Tag in den Curricula verankert werden.

## 1. Introduction

Interprofessional education (IPE) is defined as “opportunities in which two or more students from different professions learn with, from, and about each other to improve collaboration and quality of health care” [https://www.caipe.org/about-us]. The World Health Organization (WHO) recognizes IPE as a potential innovative strategy to address the global skills shortage and a need for better collaboration to meet patient needs [1]. Various studies have shown that students can improve their appreciation of other professionals and their understanding of each other's roles through IPE [2], [3], [4]. For the concrete and successful implementation of interprofessional teaching programs, international studies have identified core elements in terms of learners, teachers, processes and learning outcomes and described good practice examples [4], [5], [6]. A review paper by Reeves et al. [7] on effects on professional practice and health care found positive effects of IPE on patient satisfaction, care of patients* with diabetes, and collaboration in emergency departments, among others.

In Switzerland, there are efforts to strengthen IPE both at the political-strategic level and starting from the individual educational institutions [8]. Interprofessionality plays an important role in the overall federal strategy “Health 2020” [9] and in the learning objective catalogs of the individual professional groups [10], [11]. Within the framework of the funding program “Interprofessionality in Healthcare”, projects in the field of interprofessional education and collaboration were funded and listed by the Federal Office of Public Health (FOPH) from 2017 to 2020 [12], [13]. Although there are now interprofessional teaching programs at almost all educational institutions, many projects have been developed in relative isolation and independently of each other [14]. This could be due to the fact that the medical professions (medicine, pharmacy, etc.) are educated at universities and the health professions (nursing, physiotherapy, etc.) at universities of applied sciences and colleges of higher education. 

Research and expert reports have identified practice-based interprofessional education days as a suitable teaching format to impart interprofessional competencies [15], [16]. This paper describes the design and implementation of the 2019 Interprofessional Education Day (IPE day) as well as the qualitative evaluation results from the interviews conducted afterwards.

## 2. Project description

The IPE Day took place on October 12, 2019, in Winterthur and was organized by the Department of Health of the Zurich University of Applied Sciences (ZHAW) and the Medical Faculty of the University of Zurich (UZH). The basic concept was developed by a four-member interprofessional team of students during a summer academy of the German National Academic Foundation. Taking into account the core elements described in the two papers by Oandasan et al. [5], [6], the concept was finalized and implemented by the team of authors. Special attention was paid to the specifics of the learners, the setting-specific factors as well as the didactic concepts to achieve the learning objectives.

The goals of the IPE day were to teach students the importance of interprofessional education and collaboration, to demonstrate the roles and responsibilities of other healthcare professionals, and to have them practice taking an interprofessional history and jointly developing a treatment plan. The focus was on the interprofessional competencies of teamwork, willingness to collaborate, openness, appreciation, respect, and reflectiveness. These selected areas of competence were based on international frameworks [https://www.ipecollaborative.org/ipec-core-competencies] and national projects [https://www.zipas.ch/], [17]. 

The IPE day began with an introduction to the topic, including definitions of terms and an overview of the current evidence base on interprofessional education and collaboration in healthcare. Under the guidance of two lecturers, students then got to know each other in larger groups and used a virtual, interactive pinboard to address mutual role models and prejudices. The students were then divided into small interprofessional groups: before and after lunch, there was a case study with a simulation person (SP). Each small group was accompanied by a lecturer who, as a “facilitator”, did not primarily impart knowledge but remained in the background and facilitated learning moments [5], [18]. The two case studies followed the same procedure. After a brief case description and preliminary discussion, some of the students took a history together, worked out a treatment plan, and discussed it with the SP. During this process, they were observed by the other students. Finally, there was a feedback session, which followed the Gibbs cycle of reflection [19]. 

The first case report, “chronic regional pain syndrome”, was about a 39-year-old single mother who presented to the hospital for follow-up five weeks after a distal radius fracture. She had increasing pain as well as swelling and redness of the skin in the area of the fracture. Due to limited mobility, the patient had difficulty breastfeeding her child, doing household chores, and caring for her sick mother at the same time. The second case report, “urinary tract infection in gestational diabetes”, involved a 35-year-old mother who presented to the emergency department with increasing fatigue, malaise, burning during urination, and back pain. She was 8 months pregnant, had not attended the last check-up appointment due to her work, and blamed herself for this. 

The two case studies were followed by a final round of reflection, in which two small groups presented their most important findings to each other and the participants had another opportunity to provide individual feedback. After the closing words in the plenum, there was a barbecue to bring the day to a close.

A total of 68 students from three educational institutions (UZH, ZHAW, ETH Zurich) and seven training programs participated: human medicine (20), nursing (14), physiotherapy (12), pharmaceutical sciences (5), midwifery (5), occupational therapy (4) and doctoral program Care & Rehabilitation Sciences (8). Participants were in different years of training, which was taken into account in grouping. The eight faculty members had prior experience with interprofessional teaching. They were prepared and instructed specifically for the IPE day using a script and in a training session. 

The IPE day was evaluated qualitatively with the aim of exploring and describing the experience of the participating students and lecturers.

## 3. Methodology

The evaluation followed a qualitative, exploratory research design with semi-structured interviews. A total of 15 interviews were conducted with 14 students (two double interviews) and three lecturers. Sample recruitment was purposive to ensure that as many fields of study as possible were represented. The characteristics of the interviewees are listed in table 1 (Tab. 1).

All interviews were conducted directly after the IPE day in a quiet place and face-to-face. Under consent, all interviews were recorded, anonymized, and transcribed verbatim. Interviews lasted between 6 and 23 minutes. The interview guides were prepared considering the core elements of interprofessional teaching [5] and available frameworks [1], [20]. The goal was to address the most important aspects of the IPE day without limiting the conversations. The guiding questions were mainly aimed at the subjective experience and the experiences of the participants. Requests for improvement, role models, and elements of interprofessional teaching and collaboration were addressed. 

Data analysis followed the steps of Braun and Clarke’s thematic analysis [21] and was performed by two authors (SG, MH). After repeated reading, initial codes were formed as a basis for possible themes. After that, definite themes were specified. The analysis was performed using MAXQDA 2018 software.

According to the Ethics Committee of the Canton of Zurich, the study did not fall within the scope of the Human Research Act and therefore did not require approval (BASEC-Nr. Req-201900881).

## 4, Results

The analysis of the interview data resulted in four themes, which are illustrated by quotes below.

### 4.1. Strengths and positive aspects of the IPE day

Overall, the IPE day was experienced as very enriching by all participants. Several positive aspects were mentioned during the interviews. The participation of both medical and pharmacy students was highlighted as a major strength by the students and lecturers. 

*“At the ZHAW, the professions of midwifery, nursing, occupational therapy and physiotherapy already study in the same building and there is actually much more exchange. And now with the medical students and also the pharmacists, who actually study in two completely different buildings, it is actually also a bridging to these.” (physiotherapy student)*

*“The new thing is really the addition of the medical students. And, I have now, simply it has confirmed to me once again that that is really again like an additional gain, because that is in practice the occupational group where actually all occupational groups have many overlapping points.” (lecturer)*

Furthermore, the students considered the work in small groups and the practical case studies with simulation persons to be particularly valuable. Both the lecturers and the students saw it as a great advantage to carry out such learning moments in a “protected” training setting before implementing them in practice and to reflect on what they had experienced by means of feedback rounds together with the simulation persons.

*“And um, I found it very nice that, that you were so in the same setting. You knew that everybody studies, everybody doesn’t know everything. From that, um, I was much less afraid that I could make a mistake or forget something or something. Because I knew the others were studying too.” (student nursing)*

Finally, the informal encounters during the breaks, lunch and barbecue were also very much appreciated by all participants in order to get to know each other better and to exchange information about the respective training paths and job profiles. 

#### 4.2. Potential for improvement in the implementation of the IPE day

In the interviews, there were suggestions for improvement, especially for the introduction with the stereotypical role models. For the medical and pharmacy students, an introduction to the competencies and tasks of the other professional groups was missing. In addition, the students and lecturers criticized the technical implementation of this task by means of a virtual pinboard as time-consuming and impractical. 

*“In the beginning, we did that with the stereotypes. We just put them in the room and read them out, but afterwards not much really came out of it. And just, then it was still missing that you maybe get a short instruction, what the others learn in the study, what they can do, what they do, that you can also apply that better in the patient interview afterwards.” (medical student)*

*“I found it difficult, kind of, so until just everyone was already technically able to use that at all [...].” (lecturer)*

Despite these criticisms, the interview participants agreed that the introduction should not take too much time and found the balance between theory and practical examples very successful. In the case studies themselves, some students had difficulty imagining that such interprofessional scenarios would even occur in practice. One lecturer wished for even smaller, primarily linguistic, adjustments in the case examples.

#### 4.3. Learning effects and competence acquisition of the students through participation in the IPE day

Overall, the students found the day very instructive and were positively surprised by how much they were able to take away. Specifically, some students expressed that they were able to break down mutual prejudices and classic role models through the joint case studies. They learned to act as an interprofessional team, although this was not easy at the beginning, as there were also certain expectations of the different roles. For example, the medical students initially had the feeling that they had to take the “lead” and found it unusual to hand over this role. 

*“I think I questioned the role of the physician as leader a little bit more. We then also consciously in our role example, so the nursing took over the lead then and that was a bit strange for me at the beginning, because I’m used to doing an anamnesis alone.” (medical student)*

During the simulations and reflection rounds, the students realized the importance of an open attitude and good communication in order to learn from each other and to be able to work together effectively. Through the joint case work, the students noticed commonalities, e.g. in the collection of anamnesis, but at the same time also got to know and appreciate the competences and expertise of the other professional groups. 

*“And I mean even if you couldn't have done something specific to your profession right now, you still... everybody does the history, so the basic framework is the same for everybody.” (student midwife)*

The interprofessional exchange and collaborative case work also reinforced some students’ own roles:

*“And the other thing is what I learned, so the appreciation of occupational therapy from the medical students. It was so good, my heart, to hear that they had such an aha moment, and like, ah, you guys are really doing something mega important.” (occupational therapy student)*

The lecturers also reported that the students learned a lot in the simulations and already showed significant improvements in some interprofessional competence areas (especially teamwork and communication) in the second simulation. 

*“I also found it impressive, the difference between the first and the second simulation. So what a jump they made there, from the morning to the afternoon, and how they, the things we discussed in the reflection, that really impressed me, how quickly that worked out [...]. In the afternoon it had really been a conversation of five people with each other.” (lecturer)*

Some faculty also emphasized that there were many learning moments for themselves during the simulations and reflection sessions as well, especially in terms of group dynamics due to the participation of the medical students.

#### 4.4. Anchoring interprofessional teaching in the curriculum and implementation in professional practice

In the interviews, both students and faculty emphasized the importance of interprofessional teaching to improve subsequent collaboration and quality of patient care. They saw joint training opportunities at an early stage as a great opportunity to bring about a culture change. 

*“What’s conducive, I think, is that we’re all in training and we're all young and interested in this, and like also feel like it needs something new.” (medical student)*

The interview participants agreed that in the future there will be a need for more interprofessional teaching opportunities in the various curricula and that these must take place regularly in order to achieve a long-term learning effect. The students felt encouraged by the IPE day to actually implement what they had learned later in their professional practice. At the same time, however, they also expressed concerns that this may not yet be easy in today’s world and that it takes time.

*“Yes, I would like, if somehow possible, to bring this into practice. It’s difficult, and I don’t know how at the moment. But such a dream would be that in the future it would work the way it worked today.” (student nursing)*

*“Yes, we are still a long way from interprofessional collaboration. But, of course, I have always had respect for the other professions, but it has strengthened that even more, it has more or less reinforced the fact that the others also know a lot, and I now have the mind-set and know that too. And I might be able to integrate that into the professional life later.” (student medicine)*

## 5. Discussion

### 5.1. Summary of results

Both students and lecturers had a very positive experience of the IPE day. The participation of medical and pharmacy students, the practical case studies with simulated persons and the informal exchange during the breaks were particularly appreciated. There was potential for improvement primarily in the elaboration of role models by means of a virtual pinboard. Nevertheless, the students were able to reduce mutual prejudices and learned to know and appreciate the competencies of the other professional groups through an open attitude and good communication. All of the interviewees wished for more interprofessional courses and the students felt encouraged to apply what they had learned in their later professional practice. 

#### 5.2. Interpretation of the results and classification in the literature 

A special feature of the IPE Day, which was experienced as very profitable by all participants, was the participation of students from different professional groups and educational institutions. The inter-institutional collaboration required good and close coordination during planning and implementation, but was repeatedly described as a great strength. As described in a recommendation for action by Nock [22], the cooperation was participatory and on an equal footing on both the organizational and content levels. This procedure led to great satisfaction on the part of the team of authors and the lecturers.

The concept developed by an interprofessional team of students worked very well overall. The participants appreciated the strong practical relevance, the opportunity to practice in a protected setting and the informal exchange during the breaks. The structured reflection rounds with the involvement of the simulation participants led to a deeper examination of one's own role in the interprofessional team and the competencies of the other professional groups. In doing so, it helped to orient oneself to established feedback and reflection methods [19], [23].

As an introduction, instead of discussing mutual role stereotypes, some students would have preferred information about the knowledge and skills of the other professional groups. However, the concept envisaged that the students would work out this information themselves within the framework of the case studies and experience it in practice. From a didactic point of view, the introduction of the roles did not focus on frontal knowledge transfer, but on interactive engagement with the topic supported by digital tools [24], [25]. However, this was made more difficult by the time-intensive, virtual pinboard. 

According to their own statements, the students were able to improve the interprofessional competencies of communication and teamwork in particular through their participation in the IPE day. This was confirmed by the lecturers who witnessed the learning progress of the students between the two simulations. In addition, the students became better acquainted with and appreciated the areas of responsibility and competencies of the other professional groups in the simulations and reflection rounds, which led some to a reinforcement of their own expertise and choice of profession. 

Singer et al. [26] evaluated an IPE day with 438 students from medicine, dentistry, pharmaceutical sciences and optometry. Nagge et al. [27] studied a joint half-day educational program for 146 medical and pharmacy students. These primarily quantitative studies from Canada found statistically significant improvements in all six skill areas or 20 items of the standardized questionnaire “ICCAS” [28]. These results reinforce that such teaching formats enable an acquisition of competencies, which was subjectively described by our participants in the interviews.

#### 5.3. Outlook and further developments 

Some students felt that the joint collection of medical history in the interprofessional team was unrealistic. In professional practice, this is actually only partially practiced, for example during patient rounds in the intensive care unit [29]. In order to motivate students to implement this in their later professional life, joint training stations are important in addition to selective teaching offers, as experience from abroad has shown [30], [31], [32]. In the canton of Zurich, the “Zurich Interprofessional Training Station” (ZIPAS^®^) has been in place since the fall of 2019, where students and trainees from different health care professions care for patients as an interprofessional team under supervision [https://www.zipas.ch/]. In the context of a curriculum revision of the course of study in human medicine at the University of Zurich, interprofessionality was also defined as a focus and further IP courses are being planned [33]. At the ZHAW, there is a longitudinal interprofessional course (WIPAKO^®^) with a focus on communication and interprofessional collaboration, which students are required to complete [34]. 

However, for the successful implementation of future interprofessional collaboration, openness and corresponding structures in the health care institutions are needed in addition to the motivation of the students and the IPE offerings in the curricula. 

## 6. Conclusion

The IPE Day was successfully held for the first time in 2019. The didactic concept worked largely well. Students and lecturers particularly appreciated the cross-institutional participation of medical and pharmacy students as well as the practical implementation using case studies with simulation persons. The evaluation provided subjective evidence that the students were able to improve the interprofessional competencies of teamwork, communication, openness, appreciation, and reflectiveness, and were strengthened in their own roles. In the future, the IPE day will be anchored in the curricula together with other, longitudinal IP courses and inter-institutional collaboration will be strengthened. 

## Acknowledgement

The author team thanks Dr. Matthew J. Kerry-Krause for translating the manuscript into English.

## Competing interests

The authors declare that they have no competing interests. 

## Figures and Tables

**Table 1:**
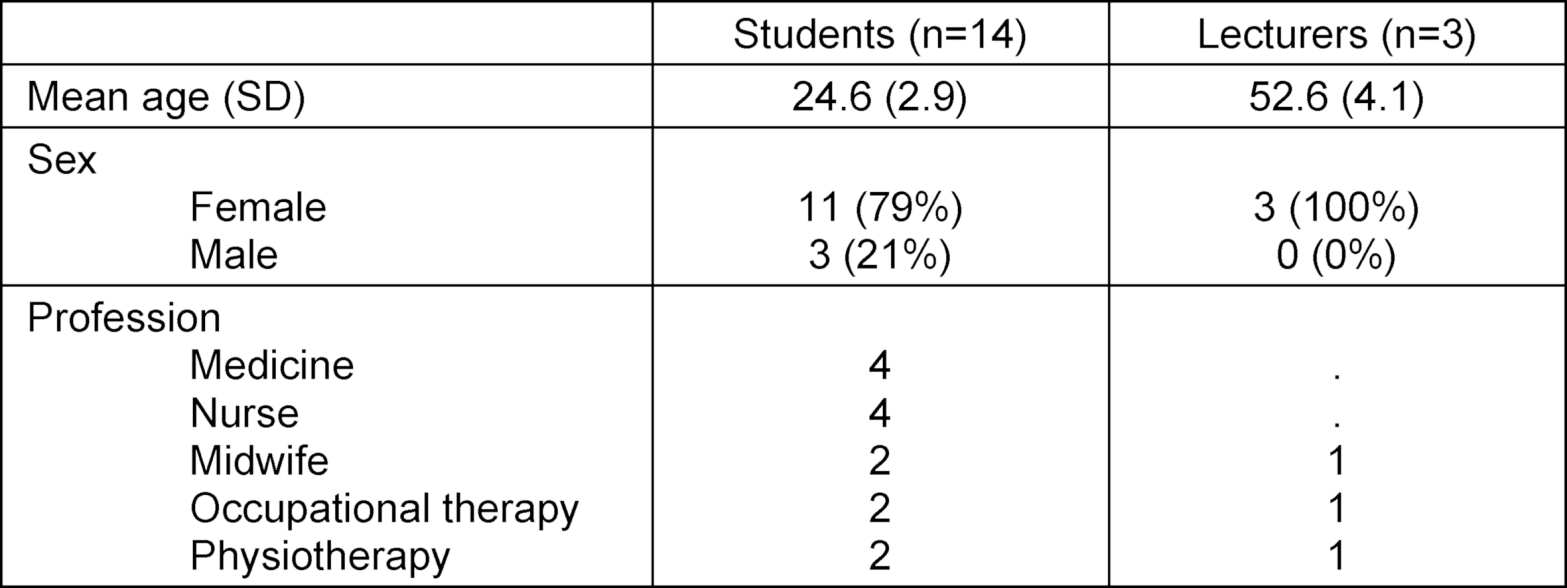
Characteristics of the Interviewees

## References

[1] World Health Organization (2021). Framework for action on interprofessional education and collaborative practice.

[2] Remington TL, Foulk MA, Williams BC (2006). Evaluation of evidence for interprofessional education. Am J Pharm Educ.

[3] Thistlethwaite J, Moran M, World Health Organization Study Group on Interprofessional Education and Collaborative Practice (2010). Learning outcomes for interprofessional education (IPE): Literature review and synthesis. J Interprof Care.

[4] Bridges DR, Davidson RA, Odegard PS, Maki IV, Tomkowiak J (2011). Interprofessional collaboration: three best practice models of interprofessional education. Med Educ Online.

[5] Oandasan I, Reeves S (2005). Key elements for interprofessional education. Part 1: the learner, the educator and the learning context. J Interprof Care.

[6] Oandasan I, Reeves S (2005). Key elements of interprofessional education. Part 2: factors, processes and outcomes. J Interprof Care.

[7] Reeves S, Perrier L, Goldman J, Della Freeth, Zwarenstein M (2013). Interprofessional education: effects on professional practice and healthcare outcomes (update). Cochrane Database Syst Rev.

[8] Walkenhorst U, Mahler C, Aistleithner R, Hahn EG, Kaap-Fröhlich S, Karstens S, Reiber K, Stock-Schröer B, Sottas B (2015). Position statement GMA Comittee–“Interprofessional Education for the Health Care Professions”. GMS Z Med Ausbild.

[9] Bundesamt für Gesundheit BAG (2021). Gesundheit2020.

[10] Michaud PA, Jucker-Kupper P, The Profiles Working Group (2016). The "Profiles" document: a modern revision of the objectives of undergraduate medical studies in Switzerland. Swiss Med Wkly.

[11] Ledergerber C, Mondoux J, Sottas B (2009). Projekt Abschlusskompetenzen FH-Gesundheitsberufe.

[12] Bundesamt für Gesundheit BAG (2021). Förderprogramm «Interprofessionalität im Gesundheitswesen» 2017-2020.

[13] Bundesamt für Gesundheit BAG (2021). Verzeichnis Modelle guter Praxis – Interprofessionalität.

[14] Ulrich G, Amstad H, Kaap-Fröhlich S (2020). Interprofessionelle Ausbildung im Schweizer Gesundheitssystem: Situationsanalyse und Perspektiven – ein Working Paper. Jahrestagung der Gesellschaft für Medizinische Ausbildung (GMA). Zürich, 09.-12.09.2020.

[15] Bundesamt für Gesundheit BAG (2021). Forschungsberichte Interprofessionalität im Gesundheitswesen.

[16] Bundesamt für Gesundheit BAG (2021). Kompetenzen zur interprofessionellen Zusammenarbeit und geeignete Unterrichtsformate.

[17] Bundesamt für Gesundheit BAG (2021). Kompetenzen zur interprofessionellen Zusammenarbeit und geeignete Unterrichtsformate.

[18] Hylin U Interprofessional education: Aspects on learning together on an interprofessional training ward.

[19] Gibbs G (1988). Learning by Doing: A Guide to Teaching and Learning Methods. Furth Educ Unit.

[20] McGill University Office of Interprofessional Education (2022). Canadian Interprofessional Health Collaborative (CIHC) framework.

[21] Clarke V, Braun V, Teo T (2014). Thematic Analysis. Encyclopedia of critical psychology.

[22] Nock L (2016). Handlungshilfe zur Entwicklung von interprofessionellen Lehrveranstaltungen in den Gesundheitsberufen.

[23] Boneberg I, Frielingsdorf A, Lippmann E, Hug B, Lippmann E, Pfister A, Jörg U, Leuenberger T (2013). Gestaltung der Beziehung zu einzelnen Mitarbeitenden. Handbuch Angewandte Psychologie für Führungskräfte: Führungskompetenz und Führungswissen.

[24] Haag M, Igel C, Fischer MR, German Medical Education Society (GMA), Committee “Digitization – Technology-Assisted Learning and Teaching”, Joint working group “Technology-enhanced Teaching and Learning in Medicine (TeLL)” of the German Association for Medical Informatics, Biometry and Epidemiology (gmds) and the German Informatics Society (GI) (2018). Digital Teaching and Digital Medicine: A national initiative is needed. GMS J Med Educ.

[25] Brezis M, Cohen R (2004). Interactive learning in medicine: socrates in electronic clothes. QJM.

[26] Singer Z, Fung K, Lillie E, McLeod J, Scott G, You P, Helleman K (2018). Interprofessional education day - an evaluation of an introductory experience for first-year students. J Interprof Care.

[27] Nagge JJ, Lee-Poy MF, Richard CL (2017). Evaluation of a Unique Interprofessional Education Program Involving Medical and Pharmacy Students. Am J Pharm Educ.

[28] Schmitz CC, Radosevich DM, Jardine P, MacDonald CJ, Trumpower D, Archibald D (2017). The Interprofessional Collaborative Competency Attainment Survey (ICCAS): A replication validation study. J Interprof Care.

[29] Stollings JL, Devlin JW, Lin JC, Pun BT, Byrum D, Barr J (2020). Best Practices for Conducting Interprofessional Team Rounds to Facilitate Performance of the ICU Liberation (ABCDEF) Bundle. Crit Care Med.

[30] Wilhelmsson M, Pelling S, Ludvigsson J, Hammar M, Dahlgren L-O, Faresjo T (2009). Twenty years experiences of interprofessional education in Linkoping--ground-breaking and sustainable. J Interprof Care.

[31] Ker J, Mole L, Bradley P (2003). Early introduction to interprofessional learning: a simulated ward environment. Med Educ.

[32] Arnold C, Berger S, Gronewold N, Schwabe D, Götsch B, Mahler C, Schultz JH (2020). Exploring early interprofessional socialization: a pilot study of student’s experiences in medical history taking. J Interprof Care.

[33] Universität Zürich (2021). Curriculumsrevision ZH Med4.

[34] Spiegel-Steinmann B, Feusi E, Wieber F, Huber M (2021). WIPAKO® Winterthur interprofessional training concept "communication and cooperation in health professions": concept, development process and implementation. GMS J Med Educ.

